# Migrant physicians’ choice of employment and the medical specialty general practice: a mixed-methods study

**DOI:** 10.1186/s12960-021-00607-x

**Published:** 2021-05-12

**Authors:** Linda Sturesson, Per J. Palmgren, Magnus Öhlander, Gunnar H. Nilsson, Terese Stenfors

**Affiliations:** 1 Department of Learning, Informatics, Management and Ethics, Karolinska Institutet, 171 77 Stockholm, Sweden; 2 Department of Ethnology, History of Religions and Gender Studies, Stockholm University, 106 91 Stockholm, Sweden; 3 Department of Neurobiology, Care Sciences and Society, Karolinska Institutet, 171 77 Stockholm, Sweden

**Keywords:** Employment, Family medicine, General practice, General practitioner, International medical graduates, Migrant physician, Specialty, Mixed methods, Qualitative, Quantitative, Primary health care, Medical specialty

## Abstract

**Objective:**

In many countries, migrant physicians (MP) tend to fill staff shortages in medical specialties perceived as low status. The aim of this study was to explore aspects that influence MPs’, with a medical degree from outside EU/EEA, choice of employment and medical specialty in Sweden, and to explore and understand a potential over-representation in general practice (family medicine), a specialty suffering from staff shortages in Sweden.

**Methods:**

A mixed-methods approach was applied. This included questionnaire data from 101 MPs training and working as medical specialists in Sweden and semi-structured interview data from four MPs specializing in general practice.

**Results:**

Regardless of specialty, the most influential aspects when choosing employment were the ability to combine work with family, to develop one´s competence, and to have highly competent colleagues. Women scored higher on some aspects related to private life and the surroundings. More than half (55%) of the respondents specialized in general practice, and more women than men. The MPs in general practice scored higher on the aspect ‘ability to have the same patients for a longer period’ than MPs specializing in other specialties. No significant difference between MP general practitioner respondents and MPs in other medical specialties was found in relation to the item ‘Was the specialty your first choice?ʼ. Aspects identified in the interviews that influenced the choice to specialize in general practice related to job opportunities, positive experiences of primary health care, working conditions, and family conditions.

**Conclusion:**

Labour market conditions such as high competition, and the time-consuming recertification process, can influence the choice to specialize in general practice as this reduces the time to become a medical specialist. We however did not find any results indicating that MPs’ decision to specialize in general practice and to work as general practitioners was any less voluntary than that of MPs who chose other specialties.

## Introduction

Many migrant physicians (MP) who arrive in their destination country intend to continue their professional careers as medical specialists. However, in some Western countries, MPs tend to fill shortages in medical specialties and sectors that are perceived as ‘low status’ [1–5]. This might suggest an inequality and imbalance in the medical specialty labour market based on the country in which a physician obtained their medical degree (cf. [4]). Further, this may indicate that MPs have fewer opportunities and decreased autonomy to choose their desired employment and specialty compared to their domestically trained peers, which can adversely impact their motivation, well-being and work–life sustainability (cf. [6, 7]). Therefore, it is imperative to explore MPs’ employment and specialty choices and the aspects that influence their decisions.

Choosing a medical specialty is an extensive individual process that often starts during medical school [7] and is often influenced by social background and family upbringing [8], gender, demographics, lifestyle compatibility, working conditions, and salaries [9]. Interest in treating specific types of patients and medical conditions also influences the choice of specialty [8–10], as do personality traits [11–13]. In addition, a specialty’s perceived status and prestige can impact the choice of specialty [8–10, 13]. Surgical specialties, for instance, afford high status and prestige, while psychiatry often does not [8, 10, 14]. Moreover, low-status specialties, such as general practice (family medicine), psychiatry and geriatrics, usually suffer from staff shortages [8–10, 13, 15].

The hierarchy of medical specialties has previously been analysed and discussed through the lens of Bourdieu’s concepts of social field, symbolic capital and habitus [8, 10]. A social field is constructed by a group of individuals and institutions with joint interests that are struggling over resources (capital) (cf. [7, 10, 16]). By gaining capital (social, cultural, economic), recognized as symbolic capital, individuals are hierarchically positioned in relation to one another [8, 10, 16, 17]. The medical labour market can be understood as a social field [8, 10] or more specifically, the medical field (cf.[18]). The medical field includes many professions in addition to physicians, such as allied healthcare workers, nurses, auxiliary nurses, healthcare assistants and untrained caregivers [10]. Primary health care (PHC) and allied health professions are usually associated with lower status [10].

The specialties in which MPs choose to train vary internationally. In Norway, MPs are more likely to become specialists than domestically trained physicians; however, domestically trained physicians are more likely to become surgeons [14]. In contrast, in Finland, MPs are less likely to specialize than domestically trained physicians, but surgery is a common specialization [2]. MPs in Finland, however, often work in PHC [2]. In the UK, MPs are more likely to work in less popular specialties, such as geriatric and psychiatry [1, 4]. Similarly, career advancement in underserved specialties might be easier for MPs in Ireland, where access to post-medical training for MPs is difficult [1]. Filling gaps in underserved areas is also an established way of shortening the recertification process in Canada [19]. This can also be seen in the high representation of MPs in PHC in the US and Finland [2, 3, 5].

Underserved areas refer to types of sector, medical specialties, and geographical locations [20, 21]. Despite the societal benefits of filling such gaps, professional development and career advancement should be equally accessible to all physicians, not assigned based on the country in which a physician received their medical education. Having less freedom to pursue a desired medical specialty and location can adversely affect well-being [6]. Additionally, if the MP workforce is concentrated in particular medical specialties, the medical specialty workforce could become unbalanced. It could also be argued that the distribution of medical specialists should reflect the diversity in the society at large.

In Sweden, 43% of medical specialists are women, and 57% are men [15]. In 2019, approximately 9800 physicians were in specialty training [15], almost a quarter (*n* = 2300) in general practice [15], making it the largest medical specialty. Physicians specializing in general practice and working as general practitioners (GPs) make up the largest employee group in PHC. In Sweden, staff shortages have been reported in PHC [8, 22] and psychiatry [8, 22]. There are possibilities that MPs with a medical degree from outside EU/EEA might work in medical specialties experiencing staff shortages in Sweden, such as general practice [23]. This study aims [1] to explore aspects that influence MPs’, with a medical degree from outside EU/EAA, choice of employment and medical specialty in Sweden and [2] to explore and understand a potential over-representation in general practice. This study will try to contribute with knowledge to help explain aspects that influence MPs’ choice of employment, why MPs may be over-represented in general practice, and if this could be interpreted as an inequality in the medical specialty labour market based on where a physician obtained their medical degree. If so, policies in order to counteract negative consequences such as decreased well-being should be developed.

## Methods

The study employed a mixed-method approach, conducted within a pragmatic and interpretative research tradition, and was part of a larger research project [24]. The current data have not been presented elsewhere. Ethical approval was received from the Regional Ethical Board in Stockholm, Sweden (2017/1717-31/5).

### Context: recertification of MPs and specialist training in Sweden

In Sweden, MPs with a medical degree from outside the EU/EEA who wants to continue to practice need to:

1. complete one of three routes to obtain the Swedish medical license: (route a) a proficiency test followed by a 6-month internship; (route b) a complementary programme for physicians (CPP), lasting 10 months; or (route c) the Swedish medical programme.
2. apply for, receive, and complete a mandatory medical internship, lasting 18 to 21 months (applies for route b and c, and was also applied on route a up until 30 June 2016). It can take years to receive a mandatory medical internship.
3. pass the exam that assess the mandatory medical internship. The test runs four times a year. The first time pass rate for MPs with a medical degree from outside EU/EEA is approximately 60% [25]. After having passed the test, the Swedish medical licence can be applied for.
4. apply for and receive a specialist training position and begin specialist training, which usually lasts 5 years.

MPs with medical degrees from outside EU/EEA are not covered by Professional Qualifications Directive 2013/55/EU, updating 2005/36/EC, in comparison to physicians educated within EU/EEA. The directive enables for EU citizens to work in other EU countries. EU-physicians are, unlike non-EU educated physicians, sometimes recruited to Sweden. During 2015 to 2019, 1.144 (187–253 yearly) non-EU educated physicians obtained the Swedish medical license [26] which is 10% of the 11.570 Swedish medical licenses that were awarded in total [27], 41% were awarded to physicians educated in another EU country of which many are Swedish citizens, and 49% to physicians educated in Sweden [26]. In 2020, approximately 41.000 physicians practised their profession [27].

### Participants

A nonprobability convenience sample of volunteer MPs with medical degrees from outside the EU/EEA were recruited via a questionnaire that was disseminated to CPP enrolees from the programme launch in 2009 and to 2017 (*N* = 497) with a response rate of 57% (*n* = 283). Respondents who indicated having a specialty training position or respondents working as specialists or as senior doctors were included in the study sample (*n* = 101).

### Data collection

Data were gathered through an electronically disseminated questionnaire (for more information, see [18]). The Swedish Board for Health and Welfare’s categorization of medical specialties was used to cluster medical specialties in the questionnaire. Specialities identified in the literature as ‘low prestige’ (general practice, geriatrics and psychiatry) were extracted and separated from their overarching categories. The following questionnaire items were used for the current study: (1) whether respondents had begun or completed specialist studies before migrating to Sweden, and if so, in which specialty; (2) whether general practice was considered a specialty in their education countries; (3) whether they were undergoing or had completed medical specialty training in Sweden (if so, in which specialty) and whether their specialty was their first choice (if not, why); (4) how they perceived the status of different medical specialties in Sweden; and (5) what aspects influenced their choice of employment. The questionnaire included closed-ended questions employing 3-point and 5-point Likert response and open-ended questions with free text answers.

To deepen our understanding of MPs’ choice of general practice and how different aspects overlap on an individual level, data were also gathered via semi-structured interviews with MPs specializing or being specialists in general practice and working as GPs. Interviewees were recruited via an email sent to MPs who had enrolled in the CPP between 2012 and 2016 (*n* = 278). Seven MPs in specialty training responded, four of whom were pursuing general practice and were included in the study (two men and two women). The interview guide included questions about the MPs’ choice of specialty and what influenced their choice, differences in the perceived prestige of specialties, and difficulties encountered in obtaining a specialty training position. The interviews lasted approximately one hour each and were audio-recorded and transcribed verbatim.

### Data analysis

The Statistical Package for the Social Sciences (SPSS) version 25.00 (IBM Corporation, Armonk, NY) was used for descriptive and inferential statistical analysis of the questionnaires. The descriptive analysis involved calculating the means and standard deviations of the quantitative variables. For the categorical variables, frequencies and percentages were generated. The data distributions were assessed visually via boxplots, by contrasting potential discrepancies among the parameters of central tendency, by evaluating the skewness and kurtosis of the distributions and by employing Shapiro–Wilk tests. Parametric statistical tests were utilized: an independent sample *T*-test for examining differences between groups and a one-way between-groups ANOVA for examine differences between three or more groups. The Chi-square (*χ*^2^) test and Cramer’s V (phi) effect size was used to compare proportions between two or more independent groups and to investigate associations between two nominal-scale variables. The Pearson correlation coefficient was calculated to test the association between the variables. *P*-values were adjusted for multiple comparisons via a Bonferroni correction of primary endpoints. *P*-values less than 0.05 were considered statistically significant for all statistical tests.

The data from responses to open-ended questions and the interview transcripts were analysed using an inductive approach to thematic analysis [28, 29]. The transcripts were first read by LS for familiarization. In another round of reading, paragraphs were highlighted on the printed transcripts, and keywords related to the manifest content were noted. In addition, thoughts and interpretations of the manifest content in relation to the researcher’s prior understanding of the research field and the entire dataset were noted. A spreadsheet was used for data sorting: interviewees were sorted by row; paragraphs, keywords and latent content (researcher’s interpretations) were sorted by column. Themes were identified by researchers and noted in another column. The findings were discussed and subject to adjustments until consensus among all researchers was reached. Despite the fact that the aforementioned steps seem consecutively ordered, the process of analysis and search for patterns was in no way linear; rather, it was iterative and recursive.

## Results

In this section, the respondents’ characteristics are presented. Aspects that influenced the respondents’ choice of employment are then addressed. A comparison of MPs in general practice and MPs in other medical specialties is then presented. The results section concludes with the interview findings.

Mean age was 41. Of the respondents, 46% were women, and 54% were men. More than one-half (55%) were either specialists or were completing their specialty training in general practice. Of the respondents who had begun or completed specialty training before migrating to Sweden, 21% were practising in their original specialty in Sweden. Of the 79% who had changed specialty, almost 60% were now practising general practice. Lastly, 24% of the respondents reported they were not practising in their first-choice medical specialty.

None of the interviewees had completed or begun specialty training before migrating to Sweden. All participants were born in the 1980s and were educated in Asia, Eastern Europe or South America. They had all migrated to Sweden between 2010 and 2013.

### Aspects that influenced choice of employment and the specialty general practice

Aspects that influenced MPs’ choice of employment and to specialize in general practice related to (1) work, private life and the surroundings, and to (2) medical labour market conditions. We also identified differences between respondents in general practice and respondents in other specialties that related to the respondents’ characteristics as presented in Table 1.

**Table 1:** Questionnaire: respondent (*N* = 101) characteristics presented as frequency and percentage (%)

	Category	In general practice in Sweden (%)	In specialties other than general practice in Sweden (%)
Position in Sweden	Undergoing specialty training or held a position as a specialist or senior doctor	55 (54)	46 (46)
Gender*	Women	31 (67)	15 (33)
	Men	24 (44)	31 (56)
Year of birth*	1970 or before	6 (55)	5 (45)
	1971–1980	31 (60)	21 (40)
	1981 or later	18 (47)	20 (53)
Region of origin (*n* = 84†)‡	Asia	26	23
	Europe	13	11
	Northern America, Latin America or the Caribbean	5	3
	Africa	4	3
Region for medical education (*n* = 96†)‡	Asia	21	27
	Europe	17	18
	Northern America, Latin America or the Caribbean	5	2
	Africa	4	3
Year of medical graduation (*n* = 94†)	2000 or before	17 (77)	5 (23)
	2001–2010	30 (51)	30 (51)
	2011 or later	4 (31)	9 (70)
General practice as a specialty in the country of medical education (*n* = 98†)	Was a specialty in country of education	22 (44)	28 (56)
	Was not a specialty in country of education	32 (67)	16 (33)
Specialty training before migrating to Sweden (*n* = 99†)	Had begun or completed specialty training	22 (56) Twenty-one indicated a specialty area. Four had begun or completed specialty training in general practice before migrating to Sweden. At least three were practising general practice in Sweden. General practice was a specialty in the country of education of 16 respondents	17 (44) Seventeen indicated a specialty area. None had begun or had completed specialty training in general practice before migrating to Sweden. Four had begun or completed specialty training in the same category as the same specialty in Sweden
	Had not begun or completed specialty training	32 (53)	28 (47)
Mean age when answering the questionnaire* (*n* = 101)		42	40

*Registry data

^†^Not every respondent answered the question. The number of respondents, therefore, differs from the total number of respondents (*N* = 101)

^‡^Respondents could indicate several countries

#### Work, private life and the surroundings

Explored aspects that influenced the respondents´ choice of employment are presented in Table 2. The most influential aspect was the ability to combine work with family. Second was the ability to develop one’s competence. Third was the competence of colleagues. The least influential aspects related to religion or other beliefs and being close to or having colleagues from one´s country of origin or education. Gender differences related to private life and the surroundings were identified (Table 2).

**Table 2:** Aspects that influence migrant physicians’ (*N* = 101) choice of employment presented as percentage-clustered categories, means and standard deviations (SD)

	Aspect	Number of answers	Little to no influences (%)	Partial influence (%)	Much or total influence (%)	Mean (SD)
Aspects related to work						
Own competence	Possibility to develop competence	98	2.0	11.2	86.8	3.9 (0.6)
	Intellectually challenging	96	8.3	29.2	62.5	3.6 (0.8)
	Challenging in terms of practical skills	96	6.3	50.0	43.7	3.4 (0.8)
	Possibility of leadership	96	50.0	31.3	18.8	2.4 (1.1)
	Possibility of doing research	97	37.1	45.4	17.5	2.6 (1.0)
	Ability to continue with the specialty started or completed before moving to Sweden	92	46.7	22.8	30.5	2.5 (1.3)
Colleagues	Colleagues are highly competent	97	2.1	21.6	76.3	3.8 (0.6)
	Swedish-trained physicians present in the workplace	97	30.9	32.0	37.1	2.9 (1.1)
	Physicians from home country present at the workplace	97	84.5	12.4	3.1	1.5 (0.8)
Patients	Diverse patient population	97	28.9	40.2	30.9	2.9 (1.0)
	Ability to have the same patients for a long period of time	97	37.1	38.1	24.7	2.7 (1.1)
Salary	High salary	97	6.2	40.2	53.6	3.5 (0.7)
Technology	The latest technology is available in the workplace	97	7.3	40.2	52.5	3.4 (0.7)
To work somewhere else	Opportunities to work in different cities/towns in Sweden	96	29.2	49.0	21.8	2.8 (1.0)
	Ability to increase opportunities to work in an EU country other than Sweden	96	43.7	37.5	18.7	2.6 (1.1)
	Ability to bring knowledge and experience from Sweden to my home country	96	45.8	27.1	27.0	2.5 (1.2)
Aspects related to private life and the surroundings						
Family	Ability to combine work with family	97	1.0	12.4	86.6	4.1 (0.7)
	Partner	83	16.8	18.1	65.1	3.6 (1.2)
	Children living at home	96	23.8	24.4	52.3	3.4 (1.2)
	Wanting to live close to children who have moved from home	51	33.4	23.5	43.1	3.1 (1.4)
	Wanting to live close to parents, siblings or other relatives	95	48.5	33.7	17.9	2.4 (1.1)
Social life	It is easy to make new Swedish friends	96	30.2	49.0	20.8	2.8*^a^ (1.0)
	Ability to live near other people from country of origin or education	96	68.8	27.1	4.2	1.8 (1.0)
Location: leisure and interest opportunities	Close to nature	97	26.8	43.3	29.9	2.9 (1.0)
	Ability to exercise/play certain sports	94	29.7	47.9	22.4	2.8 (0.9)
	Close to theatres, museums and cinemas	96	46.8	37.5	15.6	2.5*^b^ (1.0)
	Close to a university	96	47.9	38.5	13.5	2.4 (1.0)
	Close to many restaurants and cafes	96	46.9	40.6	12.5	2.4 (1.0)
	Close to a religious institution	97	78.4	16.5	5.2	1.6 (0.9)
Location: accommodation	The living area is quiet	97	10.3	21.6	68.0	3.6 (0.8)
	Quick commute to workplace	98	13.3	29.6	57.2	3.4*^c^ (0.9)
	Opportunity to pursue a career without having to move	87	19.5	29.9	50.6	3.3 (1.1)
	Good travel opportunities nearby (airport, train, etc.)	98	18.4	34.7	47.0	3.2*^d^ (1.0)
	Weather	81	45.0	25.9	29.7	2.6 (1.3)

*Statistically significant at *p* < 0.05 level

^a^Women = 3.05 (SD = 0.91) vs. Men = 2.56 (SD = 0.94); *p* = .012

^b^Women = 2.84 (SD = 0.17) vs. Men = 2.10 (SD = 0.91); *p* = 0.000

^c^Women = 3.64 (SD = 0.11) vs. Men = 3.26 (SD = 0.96); *p* = 0.027

^d^Women = 3.47 (SD = 0.92) vs. Men = 3.02 (SD = 1.01); *p* = 0.025

When comparing MPs in general practice (*n* = 55) with MPs in other specialties (*n* = 46), a significant statistical difference was found in only one aspect: ‘ability to have the same patients for a long period’ (see aspects presented in Table 2). This aspect influenced MPs in general practice significantly, compared to respondents in other specialties (M = 3.08 (SD = 0.93) vs. M = 2.18 (SD = 1.05), *p* = 0.000).

A significant association was found between gender and medical specialty (general practice or other): *p* = 0.017, phi coefficient = 0.238 where women were more likely to specialize in general practice than men (Table 1). Female respondents regardless of specialty scored higher on some aspects related to private life (Table 1).

A significant difference was found concerning respondents’ perception of the status of general practice as a specialty: MPs who specialized or were training in general practice perceived the specialty to be of lower status than physicians in other specialties perceived general practice to be (M = 1.82 (SD = 0.7) vs. M = 2.15 (SD = 0.71); *p* = 0.028).

No significant association was found between MPs in general practice and MPs in other medical specialties with regard to the variables of birth period, geographic region of origin or geographic region of medical education. However, the MPs with medical degrees from countries where general practice was not considered a specialty were more likely to specialize in general practice in Sweden than MPs with medical degrees from countries where general practice was considered a specialty: *X*^2^(1, *n* = 98) = 5.09, *p* = 0.024, phi coefficient = 0.228. No significant difference between MPs in general practice respondents and MPs in other medical specialties was found in relation to the item ‘Was the specialty your first choice?’ (Table 3).

**Table 3:** Migrant physicians’ first choice of medical specialty in Sweden

Was the specialty respondents’ first choice for specialty training	Total, *n* = 101 (%)	General practice *n* = 55 (%)	Other specialties *n* = 46 (%)
Yes	77 (76)	39 (71)	38 (83)
No	24 (24)	16 (29)	8 (17)

#### Conditions at the medical labour market

The respondents who were not working or had specialty training in their first-choice specialty, were asked to indicate their perceived reasons for why not. The respondents had applied for, but not received their first-choice specialty. Perceived reasons were often outside the MPs’ control, such as experiences of discrimination and medical labour market conditions. Ultimately, these reasons indirectly influenced MPs’ choices by redirecting them towards other specialties.

Some participants cited experiences of discrimination: ‘I did not get it [specialty training position] even though I have a PhD, but the students who were educated in the country got it right after the medical internship. All university hospitals had the same behaviour. You get no answers despite having a PhD, post-doc, and medical internship in Sweden. Highly discriminatory’. A few respondents mentioned age as an obstacle to getting a first-choice position.

Medical labour market conditions included intense market competition, which prevented respondents from getting their first-choice specialty position. Further, a lack of market contacts and references was noted as a limitation, as were a poor work environment.

Perceived reasons for not working in a first-choice specialty differed between the MPs in general practice and MPs in other specialties to some extent. For MPs in general practice, the most common reasons for not working in a first-choice specialty were the length of time it would take to land a first-choice position and a lack of contacts, respectively. For MPs in other specialties, the most common reasons for not working in a first-choice specialty were the intensity of competition and a lack of contacts and references, respectively.

### Migrant physicians’ choice to specialize in general practice: results from interviews

In-depth information about how different aspects influenced MPs’ choice to specialize in general practice was relayed through the interview stage of our study. The following themes were identified: job opportunities, positive experiences of PHC, working conditions, and family conditions.

Job opportunities are plentiful in PHC, making it easier for MPs to be employed in this area. Interviewees had been offered the opportunity to participate in specialty training at the same PHC location where they had done their mandatory medical internship. The interviewees had positive experiences of PHC during temporary work conducted before or during their internship. They mentioned enjoying working at the PHC location where they had conducted their internship prior to obtaining their Swedish medical licence. This was because they had the same colleagues and had become a team. One interviewee contrasted PHC with a hospital in which no one knows anyone and one cannot make contact with others.

Interviewees enjoyed the variety of work tasks, working with patients of different ages and diverse backgrounds, and the opportunity to establish a professional relationship with their patients: ‘You can see a little bit of everything’. One interviewee highlighted their lack of knowledge about general practice before being exposed to it: ‘I had no, not much idea about general practice before, it was more… talking, talking about cardiology, for example, neurology, and such super specialty, but not general practice as a specialty, there was no [such specialty] in [country], nor in [country]’.

Working conditions, such as salaries, working hours, and not having to be on call, were also mentioned as influential aspects in MPs’ choice to specialize in general practice. For instance, one interviewee had changed to general practice because dissatisfaction with the working conditions in another specialty. The working hours associated with general practice and working as a GP at a PHC location were also highlighted as convenient for physicians who were parents: ‘You get home at five every day, and red-letter days you are home’. Some interviewees had considered other specialties, but when their family conditions changed, their priorities did as well:

‘Then I became a mother, and all priorities changed. Considering that I have foreign background and therefore no relatives here, it is just my partner and me, and then I thought about on-call work, night work, weekend work and that was nothing I would [do]… I was not going to be the mother who was not with her children because my daughter is my first priority.’

Some interviewees initially pursued other specialties, but different circumstances changed their direction. One interviewee did not receive their first-choice specialty position, and when their second choice was not immediately available, waiting was not an option as the MP needed an income. Like the interviewee with children, this MP faced changing priorities, illustrating how influential aspects and their importance can change over time.

Other aspects of working conditions related to language: ‘If I was insecure about my language [skills], I would not have chosen general practice’, and to flexibility, as the general practice specialty and PHC workplace did allow participants to pursue their specific medical interests: ‘I have told that this is my greatest interest, so I take care of the patients with [diseases related to a specific organ]’.

## Discussion

This study aimed to explore aspects influencing MPs’ choice of employment, and to explore and understand a potential over-representation in general practice. The results are discussed related to work, private life and the surroundings, and labour market conditions. Then follows a discussion that focus on over-representation in general medicine.

### Influential aspects: work, private life and the surroundings

The results indicate that the respondents, regardless of specialty, valued and prioritized competence development, meaning they considered lifelong learning to be important. This is probably not specifically related to the respondents being MPs, but rather to the profession.

Forty-six percent of the respondents were women, and 54% were men. This reflects the gender distribution patterns among specialists in Sweden (in 2014, 43% were women and 57% men) [15]. We found that women, regardless of specialty, scored significantly higher on some aspects that related to private life and the surroundings. We also found that women were more likely to specialize in general practice than men. The results are in line with previous research, indicating an imbalance related to gender and specialty choices [15] in which ‘lifestyle factors and domestic responsibilities are much more important to women than to men’ [30]. These factors and responsibilities have though been suggested to have an increasing meaning also for men [30]. Research also suggests that women’s interest in certain specialties has decreased due to poor work climates [30].

Circumstances, such as working hours, can make it arduous to work in some specialties if, for example, one has few relatives nearby to help care for children. This might explain why, in the present study, the aspect ‘ability to combine work with family’ scored highest among all MPs, regardless of medical specialty or gender. A majority of the respondents indicated that partner and children living at home were aspects with partial to total influence when choosing employment. These aspects might have been valued differently if, for example, the respondents had not yet started a family. The respondents mean age was 41.

Scientific literature has reported that MPs hold less prestigious positions [2]. In our study, the ‘possibility of leadership’ was not a major influential aspect for the respondents when choosing employment. This suggests that respondents’ motivation to fill leadership positions is low. This may be due to competing interests, but may also relate to labour market conditions that might cause resignation [31], and decreased motivation. Attitudes might be aligned towards actual career opportunities to decrease any demotivation and frustration, thus attitudes may change over time.

### Influential aspects: medical labour market conditions

Some of the study participants mentioned experiences related to discrimination as a reason for not receiving a specialty training position in their first-choice specialty. Discrimination is a barrier to entering the labour market in general for most migrants [26–28], and this is often true for MPs [18, 32, 33]. Additionally, a devaluation of MPs competence might occur during a job seeking process [18].

Intense competition could negatively impact MPs’ careers. Research has suggested that domestically trained physicians are occasionally recruited to specialty training positions informally, via a recruitment process that begins during their medical programme [1, 8]. This can be a difficulty for domestically trained physicians, but this aspect is probably more difficult for MPs as they receive their basic medical training abroad, and therefore do not have the same opportunities in this informal recruitment process (cf. [1]). This is reflected in our results, as a lack of contacts was cited as a reason for not working in one’s first-choice specialty. Empirical studies have suggested that informal networks affect one’s career advancement opportunities in Sweden, and that this is of disadvantage for migrants [18, 31, 34]. Returning to Bourdieu´s concept of symbolic capital [16, 17], our results indicate that social capital (networks) might be an advantage when competing for certain specialty training positions. This might indirectly impact choices of employment and specialty and how the physician is subsequently positioned in the medical field. The lack of sufficient social capital among MPs can be explained by them not having developed their cultural capital in the Swedish medical field, as they had their basic medical education elsewhere.

### Over-representation in general practice

In Sweden, physicians in general practice constitute about a quarter of all specialists in training or working (cf. [15]). More than half (55%) of the respondents with a medical degree from outside EU/EEA were active general practitioners. We also found that some respondents had changed their specialty to general practice after migrating to Sweden. The respondents hence have a tendency to work in a specialty with staff shortages in Sweden, thus filling a gap in a low-prestige specialty which MPs usually also do in other countries [1, 2, 5, 19, 20, 35].

In our study, respondents in general practice indicated that it would take too much time for them to obtain a position in their first-choice specialty. Previous research has suggested that MPs adjust their career plans to pursue general practice, psychiatry and geriatrics for the sake of job availability [2, 35], rather than preferred career choice [2]. In Sweden, employers have difficulties to recruit physicians to general practice [15]. Applying for a medical specialty training position in a specialty suffering from staff shortages likely accelerates career advancement. This may be desirable as MPs with medical degrees from outside the EU/EEA in Sweden must undergo a time-consuming recertification process before they can proceed to specialist training, regardless of any specialty training they received before migrating to Sweden. In general, MPs are also older than domestically trained physicians at the same stage in their career [14, 25, 36]. Age in combination with labour market conditions which may include an informal recruitment process that begins in medical school, and feelings of discrimination, may help explain the tendency of MPs to work in specialties with staff shortages (cf. [1, 2, 5, 19]). Labour market conditions might limit freedom to choose and decrease motivation to pursue a certain specialty. This can lead to stress, dissatisfaction and decreased well-being [6, 7], and if change of specialty, a waste of competence. The extent of free choice can be discussed by drawing parallels to motivation (cf. [6]). Accordingly, conditions on the labour market, as well as circumstances in life, that might limit an individual’s freedom of action may be internalized and consequently accepted, turned to intrinsic motivation, culminating in the perception that one’s choices are free (cf. [6]).

Our interpretation of the findings is that the respondents in our study did not perceive their choice to practice general practice as more involuntary than respondents in other specialities. Our findings demonstrated no differences between respondents in general practice and respondents in other medical specialties regarding whether they were practising in their first-choice specialty. We also found that respondents in general practice scored significantly higher on ‘ability to have the same patients for a longer period’. Previous research has shown that an essential part of working as a GP is to develop a continuous relationship with patients [37]. Not surprisingly, this aspect also influences domestically trained physicians’ specialty choice [7].

Our analysis of the interviews revealed that previous positive experiences at PHC locations and changes in family conditions were shown to influence the interviewees’ choice of specialty and their attitudes towards their specialty. Their choice to practise general practice was described as closely connected to their positive experiences of working in PHC and the accompanying working conditions. Aspects that influence MPs’ choice of employment and specialty, and the importance of these aspects, might thus change as time passes and life circumstances change, probably regardless of specialty. Our results show that life situation affects individuals’ consideration of working conditions. As described, MPs are in general older than domestically trained physicians, and if having families, one might choose to pursue with specialties considered as lifestyle-friendly (cf. [10]), such as general practice. Other specialties that have been described as more lifestyle-friendly include public health medicine and dermatology [10]. This might also correlate with gender if, for example, women are more responsible for dropping off and picking up children from daycare. As mentioned in the beginning of the discussion, women respondents more often specialized in general practice than men, and previous research shows an imbalance regarding gender and specialty choices, which can relate to domestic responsibilities and lifestyle factors [15].

Based on these results, we suggest that labour market conditions on societal and organizational levels and circumstances of being a MP, and influential aspects on an individual level that relate to work, private life and the surroundings, and motivation are intertwined and might lead to over-representation in general practice and PHC.

We found that MPs in general practice valued their specialty less than medical specialists in other specialities valued general practice. Our analysis showed that the MPs in general practice were not in the specialty for its status. These results are congruent with research that has shown that domestically trained physicians in general practice also rank the specialty as having low status [38]; that they are aware of their specialty’s low status but still are satisfied with their choice [8]. Not all physicians strive for high-prestige specialties, like surgery [8]. As previous international research has suggested that specialties are ranked, with general practice considered low, we suggest that some ideas within the medical field, such as the hierarchy of medical specialties, might be transnational and thus not bound by national borders (cf. [14]).

Research suggests that those who work in the PHC sector have lower prestige in the medical field [10], and that specialties with a majority of women usually have low status [13]. The present study found that more than half of the respondents had specialized in general practice, the majority in PHC. In addition, women respondents were more likely to specialize in general practice than respondent men. A negative consequence of labour gaps being filled by a certain group is an imbalanced professional community. This can create divisions in the medical field based on country of medical education, or country of origin, or gender. An imbalance might produce, maintain or reproduce differences in the statuses of specialties, thereby reinforcing other types of negative differences, such as devaluation of competence based on country of origin or education, but also based on gender.

### Strengths and limitations

A limitation of the study is that only a few physicians specializing in general practice volunteered to be interviewed which may influence the qualitative results. A small sample of interviewees limited a selection based on variation regarding age, country for medical education and country of origin, and whether general practice was a specialty or not in the country of medical education. Therefore, the interesting finding revealing that respondents with medical degrees from countries where general practice was not a specialty were more likely to specialize in general practice in Sweden, was not explored in-depth. The mixed-methods design of the study was however a strength, as the interview data provide more in-depth illustration of the questionnaire data. In addition, the breadth of the questionnaire data compensated for the small sample. We also had a well-defined population for the questionnaire as all of the CPP participants in Sweden between the admission years 2009–2017 were eligible (*N* = 497). The questionnaire respondents (*n* = 283), were in comparison to the population in question, representative regarding gender, age, CPP admission year, university for the CPP, and frequency of obtained Swedish medical licence. The population mean age was 40, the questionnaire respondents 39 (*n* = 283), and respondents in specialist training or being specialists 41 (*n* = 101). The respondents in the current study were slightly over-represented in the earlier admission years which probably reflects the process and development of becoming a medical specialist in Sweden. Therefore, we consider our sample to be representative. Another strength of the study was that the questionnaire was digitally disseminated which enabled a dynamic approach where follow-up questions were asked only when relevant, to reduce any survey fatigue.

### Implications for research and healthcare

We recommend future studies exploring aspects that influence employment and specialty choices of domestically trained and EU-trained physicians, in order to do comparison with our study participants. Such studies would provide increased information about if and how the same medical specialty labour market may (re)produce different conditions depending on where a physician acquired their medical degree. Knowledge of similarities, on the individual level, across the different groups could be used for employers to attract physicians of diverse backgrounds to medical specialties or geographical regions where there are staff shortages, and to address inequality. Results could then be used to address needs to create sustainability in work life. Further research could be undertaken to identify advantages and disadvantages regarding the tendency for MPs to fill staff shortages in, for example, general practice, as well as the related implications for MPs, patients, and society at large. Future studies could explore how skills and knowledge of MPs specialized in general practice with a specialist training from another country can be utilized. These studies might investigate how the specialty can be enriched by MPs’ additional expertise and knowledge, as such enrichment may relieve other specialist areas, even if only to a small degree.

## Conclusion

Regardless of specialty, study participants valued being able to combine work with family and having opportunities to develop competences high when choosing employment. Women scored higher on some aspects related to private life and the surroundings. MPs are generally older than their domestically trained peers and have to participate in a time-consuming recertification process. Specializing in a specialty suffering from staff shortages such as general practice may reduce time to become a specialist. The present study showed an over-representation of respondents in general practice. We found that women respondents were more likely to specialize in general practice than men.

When MPs encounter difficulties reaching certain specialties, they may instead choose to fill staff shortages in others. However, we did not find any indications that respondents’ choice to specialize in general practice was any less voluntary than that of respondents in other specialities. MP general practitioner interviewees had positive experiences of working at PHC, and had their priorities changed which had influenced their choices. Influential aspects might evolve with life circumstances over time. The choice to specialize in general practice amongst MPs is complex, and involves different aspects that could be related to societal, organizational and individual levels, and these aspects interact. However, filling staff shortages creates a risk that the MPs’ competences will be underutilized, and that the medical field becomes segmented, which have implications for both healthcare and future research.

## Data Availability

The dataset generated and analysed in the course of the current study is not publicly available due to ethical restrictions. The data are protected by confidentiality rules pursuant to the Swedish Public and Privacy Act, which means that no unauthorized person can access the data. This is to protect the participants’ confidentiality and privacy. Any questions regarding the data can be emailed to linda.sturesson@ki.se.
